# Access to Healthcare, Easiness to Receive Prescription Drugs, and CAHPS measure by Social Vulnerability among Medicare Beneficiaries

**DOI:** 10.1002/alz70860_101656

**Published:** 2025-12-23

**Authors:** Teagan Maguire, Jie Chen

**Affiliations:** ^1^ University of Maryland, College Park, MD, USA; ^2^ University of Maryland School of Public Health, College Park, MD, USA

## Abstract

**Background:**

Self‐reported healthcare quality often reflects older adults’ health experiences and has been linked to improved clinical outcomes. However, limited evidence exists on the relationship between an area's social vulnerability level and patient‐reported healthcare quality scores and access to care and prescribed medicine. This study examined the relationship between social vulnerability and patient‐reported healthcare quality measures among Medicare beneficiaries, focusing on overall healthcare quality, ease of accessing needed care and medications, and the physician‐patient relationship. Findings from this study aim to provide actionable insights for policymakers to improve healthcare quality and equity for older adults in socially vulnerable communities.

**Method:**

We analyzed pooled data from the 2018, 2019, 2021, and 2022 Consumer Assessment of Healthcare Providers and Systems surveys, linked with the Social Vulnerability Index (SVI) using beneficiaries’ county of residence.

**Result:**

We found lower healthcare quality scores in more socially vulnerable areas. Among Medicare FFS beneficiaries, individuals in higher SVI categories (SVI3‐SVI4) reported significantly lower odds of favorable healthcare ratings, ease of accessing care and treatments, and consistent positive experiences with their healthcare provider. They were also less likely to report ease of obtaining prescribed medications. Similar trends were observed among MA beneficiaries, with those in SVI3‐SVI4 areas reporting lower odds of high healthcare ratings, ease of accessing care, and obtaining prescriptions.

**Conclusion:**

These findings underscore persistent disparities in self‐reported healthcare quality among Medicare beneficiaries living in socially vulnerable areas. Improving access to prescription medications and healthcare services in these communities is critical to promoting equitable outcomes.

